# Danger on the plate: human health risks derived from the consumption of angular angelshark (*Squatina guggenheim*) meat in southeastern Brazil

**DOI:** 10.3389/ftox.2025.1645858

**Published:** 2025-10-17

**Authors:** Amanda Pontes Lopes, Rebeca Dias de Souza Coutinho, Tatiana Dillenburg Saint’Pierre, Rachel Ann Hauser-Davis

**Affiliations:** ^1^ Laboratório de Avaliação e Promoção da Saúde Ambiental, Instituto Oswaldo Cruz Fundação Oswaldo Cruz, Rio de Janeiro, Brazil; ^2^ Programa de Pós-Graduação em Biodiversidade e Saúde, Instituto Oswaldo Cruz, Fundação Oswaldo Cruz, Rio de Janeiro, Brazil; ^3^ Laboratório de Espectrometria Atômica (Labspectro), Departamento de Química, Pontifícia Universidade Católica do Rio de Janeiro (PUC-Rio), Rio de Janeiro, Brazil

**Keywords:** chondrichthyes, chemical pollution, elasmobranchii, food safety, food contamination

## Abstract

**Introduction:**

Shark and ray species are particularly vulnerable to pollutant bioaccumulation, including metals and metalloids, due to their k-strategist characteristics and mid–high trophic level. The angular angelshark (*Squatina guggenheim*) is a benthic and highly endangered species distributed from southeastern Brazil to southern Argentina. Despite being threatened with extinction and banned from marketing and consumption in Brazil, it is still widely consumed in several states. However, studies addressing metal and metalloid contamination in the meat of this species have not yet been conducted in Brazil.

**Objectives:**

This study aimed to determine metal and metalloid contamination levels in *S. guggenheim* and to assess human health risks associated with its consumption by infants, children, teenagers, and adults, considering consumption frequencies ranging from one to five times per week.

**Results:**

etal and metalloid concentrations in muscle tissue were generally lower than those reported for other benthic Squatinidae species, except for Pb and Rb. Several elements were reported for the first time in this species, providing baseline data. Although a favorable Se:Hg balance suggested a potential protective effect, multiple toxic and potentially toxic elements were detected, posing significant human health risks, particularly for infants and children. Arsenic concentrations exceeded Brazilian safety limits, while Ti and Rb were present at relatively high levels, despite the absence of established regulatory thresholds. Estimated intake values and non-carcinogenic risk indices (THQ and HI) surpassed safety limits for As (notably the inorganic As 10% fraction), Cu, MeHg, and Se in different scenarios, with As exceeding the acceptable threshold by up to 415 times, even under low-frequency consumption. Carcinogenic risk (CR) estimates indicated concerning levels for As and Pb across age groups.

**Conclusions:**

The results highlight significant human health risks associated with the consumption of *S. guggenheim*, particularly for vulnerable populations such as infants and children. These findings highlight the urgent need for continuous monitoring of benthic elasmobranchs and reinforce caution regarding their consumption.

## Introduction

Despite the vital importance of the Earth’s oceans for life, these environments are increasingly suffering due to anthropogenic activities. Chemical pollution owing to direct domestic sewage and industrial waste inputs without adequate treatment combined with other chemical contaminant sources, including oil spills and dredging events, has spread widely throughout marine environments, significantly contributing to ocean degradation (UN, 2017), especially affecting organisms that inhabit coastal areas, where anthropogenic activities are most concentrated (IBAMA, 2002; Lacerda et al., 2006). In this sense, metals and metalloids are among the most dangerous aquatic pollutants, mainly due to their persistence, potential toxicity, and ability to bioaccumulate and, in some cases, biomagnify in exposed organisms (Censi et al., 2006; Miller et al., 2002). The toxicity of many elements is directly associated to their chemical species, *i.e.*, oxidation state, in addition to environmental physicochemical factors, such as temperature and salinity (Akter et al., 2005).

Marine resources are essential in human diets worldwide, and even with the advancement of fish farming, traditional fisheries are still paramount for the food supply of human populations (FAO, 2024). In 2022, for example, fishery activities captured approximately 92.3 million tons of aquatic organisms: 11.3 million from inland waters such as rivers and lakes and 81 million from the oceans (FAO, 2024). In this context, marine organisms frequently consumed by humans, such as bony and cartilaginous fish, along with invertebrates such as shrimp, squid, and crabs, represent one of the main human metal and metalloid exposure routes (Arcagni et al., 2018; Taylor and Calabrese, 2018).

Sharks and rays occupy intermediate to upper food chain positions and are, thus, particularly subject to chemical contaminant accumulation and amplification (Navia et al., 2016). Certain biological characteristics, such as slow growth, long life, and coastal habits displayed by several species during certain life stages significantly increase their exposure to chemical pollutants (ICMBio and CEPSUL, 2023). This is even more noteworthy in the case of benthic species, which remain in direct contact with sediment throughout their lives, where various contaminants, including metals and metalloids, are deposited and accumulated (Navia et al., 2016; Chiaia-Hernández et al., 2022; Özşeker et al., 2022; Wang et al., 2025).

*Squatina guggenheim* (Marini, 1936), a lecithotrophic viviparous species popularly known as the angular angelshark, with a reproductive cycle of approximately 3 years, is distributed in the continental shelves of the southwest Atlantic Ocean, from the state of Rio de Janeiro, Brazil, to the Gulf of San Matias, Argentina, also including Uruguay, inhabiting sandy and muddy substrates at depths of 10–80 m (Ebert et al., 2013). This species remains motionless on the seabed during the day and becomes active at night, making it highly susceptible to fishing exploitation by bottom gillnets (Ebert et al., 2013; Oddone et al., 2018; Gadig, 2023), resulting in declining populations, with a reduction of up to 85% in certain areas, such as southern Brazil (Vooren and Klippel, 2005; Oddone et al., 2018), leading to its current classification status as “endangered” (EN) by the International Union of Conservation of Nature (IUCN) Red List (Oddone et al., 2018).

Although highly threatened with extinction, the angular angelshark is widely consumed by humans in many areas worldwide, including Brazil. Given the absence of human health risk assessments for the species in Brazil, the main objective of this study was to quantify the concentrations of metals and metalloids in angular angelshark specimens captured off the coast of Rio de Janeiro and evaluate the potential risks associated with their consumption by humans using toxicological risk indices. This study hypothesized that, due to the species’ benthic habits, trophic level, and proximity to anthropogenically influenced coastal environments, angular angelsharks would exhibit elevated concentrations of several elements at levels potentially exceeding those recommended by food safety guidelines, thus representing a risk to human consumers.

## Methodology

### Angular angelshark sampling and processing

**FIGURE 1:**
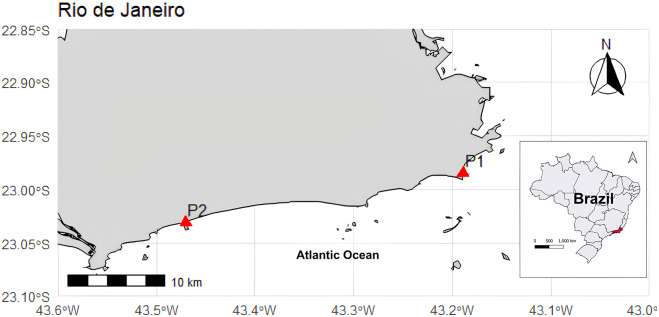
Map of the angular angelshark (*Squatina guggenheim*) sampling sites in Rio de Janeiro, southeastern Brazil. P1: Copacabana and P2: Recreio dos Bandeirantes.

Forty-two angular angelshark specimens were incidentally caught between April 2021 and December 2022 by artisanal fishers from two fishing colonies in the state of Rio de Janeiro, Brazil, namely Recreio dos Bandeirantes (n = 42) and Copacabana (n = 1) (Figure 1), totaling 15 male and 27 female specimens (eight adult and 33 juvenile specimens). Samplings were authorized by the Chico Mendes Institute for Biodiversity Conservation, SISBIO license no. 77310-1. The specimens were identified to the species level according to Gomes et al. (2020), sexed, weighed (kg), and measured (total length, TL, in cm). Muscle samples were then removed and frozen at −30 °C until further processing.

### Metal and metalloid determinations

Approximately 100 mg of each muscle sample were weighed after thawing, transferred to sterile 15-mL Falcon tubes, mixed with 1.0 mL of double-distilled nitric acid (67% v/v), and heated at 100 °C for approximately 5 h in the closed tubes to avoid the loss of volatile elements (USP, 2013). After cooling, the samples were diluted with Milli-Q water (resistivity >18.0 MΩ cm^−1^) for the elemental analysis using a NexIon 300X inductively coupled plasma mass-spectrometry apparatus (PerkinElmer, United States). Calibration was performed with a mixed standard solution (Merck IV, Sigma Aldrich) for curve preparation, using Rh as an internal standard added continuously online from a 20 mg L^−1^ solution.

Metal and metalloid readings were taken five times for each sample. The coefficients of determination (*R* ^2^) of the analytical curves were greater than 0.995 for all elements. The instrumental Limits of Detection (LOD) and method Limits of Quantification (LOQ) were calculated according to the Brazilian National Institute of Metrology, Quality, and Technology guidelines (INMETRO, 2020) as LOD = (3 × SD)/slope of the line and LOQ = (10 × SD × DF)/slope of the line, where SD represents the standard deviation of the ratio between the analytical and internal standard signals obtained from 3 and 10 blank samples, respectively, and DF is the sample dilution factor. Recoveries obtained for the certified reference materials (CRM) DORM-2, BCR-668, BB-422, and NIST-2976 (all analyzed in triplicate) are detailed in the Supplementary Material (Supplementary Tables S1, S2), alongside the LOD and LOQ values for each element. Recoveries ranged from 70% to 123%, which is consistent with internationally accepted performance ranges for elemental analysis in biological tissues and considered adequate for this assessment. Comparison between the observed and certified values was performed using Student’s t-test (α = 0.05). No statistically significant differences were found (p > 0.05) for most elements, indicating good agreement with certified values. However, two elements, Ag and As, showed p-values below 0.05 in one of the CRMs, with recovery rates of 49% and 123%, respectively. Despite this, both elements were also determined in a second CRM, where no significant differences were observed (p > 0.05). Therefore, although discrepancies were detected in a single material, the confirmation of acceptable agreement in an independent CRM supports the reliability of the method for these elements. Each analytical batch included both procedural blanks and certified reference materials to ensure ongoing quality control. Procedural blanks were systematically analyzed to detect potential contamination, with measured concentrations consistently below method LOD, confirming the integrity of the analytical process. The combined use of blanks and matrix-matched certified reference materials ensures the accuracy and trueness of the determinations, supporting the reliability of the applied methodology.

### Statistical analyses

All statistical analyses were carried out using either Excel software v. 2501 or GraphPad Prism software v. 10.4.0. A data normality analysis was performed using the Shapiro–Wilk test. As nonparametric data distributions were observed, the Spearman correlation test was applied to examine potential associations between quantitative variables. A p-value of 0.05 was established as significant. Metal and metalloid concentrations below elemental LOQ were not used in any of the calculations.

### Human health risk assessments

Metal and metalloid concentrations in angular angelshark muscle tissues were compared to Brazilian and international (Food and Agriculture Organization-FAO and European Commission-EU) guidelines regarding maximum permissible intake limita for humans. The following indices were calculated: Estimated Daily Intake (EDI), Estimated Weekly Intake (EWI), Selenium Health Benefit Value (Se-HBV), Modified Selenium health Benefit Value (HBVSe), Target Hazard Quotient (THQ), Hazard Index (HI), target cancer risk (TCR), and Carcinogenic Risk (CR) and Target Cancer Risk (TCR), analyzed for angular angelshark meat consumption scenarios of 1, 2, 3, 4, and 5 times per week (Table 1).

**TABLE 1:** Human health risk indices used to estimate potential risks associated to the consumption of angular angelshark (*Squatina guggenheim*) meat in Rio de Janeiro, Southeastern Brazil.

Index	Formula	Used variables	Range and limit	Reference
Estimated Daily Intake (EDI) (µg kg^−1^ day^−1^)	EDI = (C × FIR)/BWa	C is the concentration of a certain metal or metalloid detected in the analyzed foodstuff (mg kg^−1^). FIR is the daily food intake rate (26.71 g day^−1^ in Brazil) (Sonoda and Shirota, 2012), and BWa is the reference body weight for each evaluated age group (infants, children, teenagers, and adults).	• Infants: Cd < 0.00083 mg kg^−1^ day^−1^ bw; Cu < 1 mg kg^−1^ day^−1^ bw; MeHg < 0.0016 mg kg^−1^ day^−1^ bw; Se < 0.06 mg kg^−1^ day^−1^ bw; and Zn < 7 mg kg^−1^ day^−1^ bw. • Children: Cd < 0.00083 mg kg^−1^ day^−1^ bw; Cu < 3 mg kg^−1^ day^−1^ bw; MeHg < 0.0016 mg kg^−1^ day^−1^ bw; Se < 0.13 mg kg^−1^ day^−1^ bw; and Zn < 13 mg kg^−1^ day^−1^ bw • Teenagers: Cd < 0.00083 mg kg^−1^ day^−1^ bw; Cu < 4 mg kg^−1^ day^−1^ bw; MeHg < 0.0016 mg kg^−1^ day^−1^ bw; Se < 0.25 mg kg^−1^ day^−1^ bw; and Zn < 22 mg kg^−1^ day^−1^ bw • Adults: Cd < 0.0008 mg kg^−1^ day^−1^ bw; Cu < 5 mg kg^−1^ day^−1^ bw; MeHg < 0.0016 mg kg^−1^ day^−1^ bw; Se < 0.30 mg kg^−1^ day^−1^ bw; and Zn < 25 mg kg^−1^ day^−1^ bw.	EFSA, (2006); FAO/WHO, (1995); USEPA, (2000)
Estimated Weekly Intake (EWI) (µg kg^−1^ week^−1^)	EWI = EDI × 7	The EDI is calculated as the estimated daily intake multiplied by 7.	• Infants: Cd < 0.0058 mg kg^−1^ day^−1^ bw; Cu < 7 mg kg^−1^ day^−1^ bw; MeHg < 0.0112 mg kg^−1^ day^−1^ bw; Se < 0.42 mg kg^−1^ day^−1^ bw; and Zn < 49 mg kg^−1^ day^−1^ bw. • Children: Cd < 0.0058 mg kg^−1^ day^−1^ bw; Cu < 21 mg kg^−1^ day^−1^ bw; MeHg < 0.0112 mg kg^−1^ day^−1^ bw; Se < 0.91 mg kg^−1^ day^−1^ bw; and Zn < 91 mg kg^−1^ day^−1^ bw • Teenagers: Cd < 0.0058 mg kg^−1^ day^−1^ bw; Cu < 28 mg kg^−1^ day^−1^ bw; MeHg < 0.0112 mg kg^−1^ day^−1^ bw; Se < 1.75 mg kg^−1^ day^−1^ bw; and Zn < 154 mg kg^−1^ day^−1^ bw • Adults: Cd < 0.0058 mg kg^−1^ day^−1^ bw; Cu < 35 mg kg^−1^ day^−1^ bw; MeHg < 0.0112 mg kg^−1^ day^−1^ bw; Se < 2.1 mg kg^−1^ day^−1^ bw; and Zn < 175 mg kg^−1^ day^−1^ bw.	EFSA, (2006); FAO/WHO, (1995); USEPA, (2000)
Modified Selenium Health Benefit (HBVSE)	HBVSe = ([Se − Hg]/Se) × (Se + MeHg)	Se, Hg, and MeHg correspond to the concentrations of these elements in the investigated foodstuff.	• Negative values indicate Hg in excess, without any protective effect against Hg toxicity. • Positive values indicate Se in excess, leading to a protective effect against Hg toxicity.	Ralston et al. (2016)
Modified Selenium Health Benefit Value (SE-HBV)	Se-HBV = (Se × [Se/Hg]) − (Hg × [Hg/Se])	Se and Hg correspond to the concentrations of these elements in the investigated foodstuff.	• Negative values indicate Hg in excess, without any protective effect against Hg toxicity. • Positive values indicate Se in excess, leading to a protective effect against Hg toxicity.	Ralston et al. (2016)
Modified Selenium Health Benefit Value (THQ)	THQ = [(EFR × Ed × FIR × C)/(RfD × BWa × ATn)] × 10^−3^	EFR is the exposure frequency to a certain metal or metalloid. Ed is the exposure duration (calculated as the person’s age minus the period that the person was not exposed. established as 6 months). FIR is the foodstuff intake rate (g day^−1^). C is the wet weight concentration of a certain element in the given food item. RfD is the reference oral dose of the determined element (μg g^−1^ day^−1^) (EPA, 2002). BWa is the reference body weight of each evaluated age group (infants, children, teenagers, and adults). ATn is the average exposure time (365 days × ED), and 10^−3^ is a conversion factor unit. The RfDs used for each element were as follows: Ag 5 × 10^−5^ μg g^−1^ day^−1^, iAs 3 × 10^−4^ μg g^−1^ day ^−1^, Cd 1 × 10^−3^ μg g^−1^ day ^−1^, Cr 3 × 10^−3^, Cu 5 × 10^−2^ μg g^−1^ dia^−1^, 1 × 10^−4^ μg g^−1^ day ^−1^, 0.14 μg g^−1^ day ^−1^, 3.6 × 10^−3^ μg g^−1^ day ^−1^, Se 5 × 10^−3^ μg g^−1^ day ^−1^, V 9 × 10^−3^ μg g^−1^ day ^−1^, and Zn 0.3 μg g^−1^ day ^−1^ (EPA, 2002).	• THQ < 1: no carcinogenic effects are expected; • THQ > 1: possibility of adverse health effects.	USEPA, (1989), USEPA, (2010)
Hazard Index (HI)	$\text{HI}=\sum_{N=1}^{i}{\text{THQ}}_{n}$	Used to evaluate potential non-health effects from simultaneous exposure to multiple chemical substances.	• HI < 1: no carcinogenic effects are expected; • HI > 1: possibility of adverse health effects.	
Carcinogenic Risk (CR)	RC = CSF × EDI	CSF is the cancer slope factor according to the USEPA, and EDI is the estimated daily intake. The following CSF values (mg kg ^−1^ day ^−1^) were used: As = 1.5, Cd = 10–^3^, and Pb = 357 × 10–^5^ (USEPA, 2000; EPA, 2017; Erasmus et al., 2022).	• CR > 10^–5^: unacceptable risk.	USEPA, (2000); Erasmus et al. (2022)
Target Cancer Risk (TCR)	TCR = [(EFR × Ed × FIR × C × CPSO)/(BWa × ATc)] × 10-3	CPSO, the oral cancer slope factor, for inorganic arsenic (iAs), Cd, and Pb, are 1.5 mg kg^−1^ day^−1^, 3.8 × 10^−3^ mg kg^−1^ day^−1^, and 357 × 10^−5^ mg kg^−1^ day^−1^, respectively (USEPA, 2012). This index was calculated for As using As 1% and As 10% levels in angular angelshark meat (FSANZ, 2019), due to varying inorganic As levels in elasmobranchs, usually ranging from 1% to 10%.	• 10^–6^ < TCR < 10^–4^: acceptable; • TCR > 10^–4^: unacceptable risk.	Antoine et al. (2017); USEPA, (2012)

Three risk scenarios concerning angular angelshark muscle consumption were simulated, using the minimum, mean, and maximum concentrations determined for each element, covering the least- and most-contaminated specimens. All parameters were analyzed under these conditions for angular angelshark meat consumption scenarios of 1, 2, 3, 4, and 5 times per week. All indices were calculated for four age groups and for both sexes: infants (7 months–2 years of age, as fish consumption is recommended from 6 months, when foods other than breast milk are to be offered) (SBP, 2019), children (3–11 years old), teenagers (12–18 years old), and adults (19–75 years old). The body weights used for each age class followed UNIMED (2021) for individuals up to 18 years of age and the Brazilian Institute of Geography and Statistics (IBGE, 2024) for those aged from 19 to 75.

It is important to note that Hg calculations were carried out using 90% of total Hg concentrations as the organic form of this metal, methylmercury (MeHg), is the most toxic form of Hg and estimated as accounting for 90% of the total Hg content in fish muscle. Furthermore, two inorganic (toxic) As contamination scenarios were applied, 1% and 10%, as inorganic As levels in elasmobranchs have been reported as ranging between these values (FSANZ, 2019; Marengo et al., 2018; Souza-Araujo et al., 2021), although significant variations have been noted due to differences in life stage, diet, and trophic niche, all of which significantly affect As levels in fish.

Finally, to determine potentially protective effects of essential elements against toxic or potentially toxic elements, molar ratios of essential elements against toxic or potentially toxic element were calculated. This was carried out by converting the concentrations of each element to mol g^−1^. These values were then standardized by dividing the number of moles of the essential element by the number of moles of the toxic element. When this molar ratio is greater than 1, it indicates a potential protective effect of the essential element, suggesting that its presence may mitigate the adverse effects caused by the toxic or potentially toxic element.

## Results and discussion

### Angular angelshark characteristics

The angular angelshark is distributed along the continental shelf of the Southwest Atlantic, between Rio de Janeiro and the Gulf of San Matias, in northern Patagonia (Awruch et al., 2008). It is a relatively small demersal shark, measuring a maximum of 95 cm in TL and inhabiting depths ranging from 10 to 80 m (Ebert et al., 2013). Angular angelsharks, both male and female, begin their reproductive activities at approximately 4 years old, between 73 and 80 cm TL (Vaz and De Carvalho, 2013). The species is lecithotrophic viviparous, in which embryos develop from the nutrients present in oocyte yolk (Oddone et al., 2018). Females require approximately 2 years to prepare for gestation, which lasts approximately 12 months, totaling a 3-year reproductive cycle (Vooren and Klippel, 2005). During this process, female species produce approximately 300 g of yolk per gestation, representing about 10% of their body weight (Vooren and Klippel, 2005). The species is nocturnal, actively foraging at night, remaining motionless on the sea floor during the day, making it highly susceptible to gillnets and bottom trawl fisheries (Standora and Nelson, 1977). In Brazil, angular angelsharks are not usually targeted by fishing, captured mainly as bycatch, and although its marketing is officially prohibited (MMA and ICMBio, 2014), the species is still found in markets sold for human consumption in Brazil (Almerón-Souza et al., 2018). The species preys on bony fish, cephalopods, and finfish, among others (Vögler et al., 2003), occupying a relatively high trophic level of 4.4 ± 0.80 and, thus, categorized as a tertiary consumer (Froese and Pauly, 1999) and highly susceptible to chemical contamination.

### Angular angelshark biomorphometric data

Reporting morphometric and biometric data for fish species consumed by humans and analyzed for chemical contaminants is important when assessing potential human health risks, as metal accumulation is strongly influenced by biological factors such as size, weight, age, and sex, with larger and older individuals generally exhibiting higher concentrations due to longer exposure times and bioaccumulation processes. Furthermore, consumers often prefer fish within specific size ranges, so contamination data can, therefore, be directly related to the individuals most likely to be consumed. Additionally, biometric information enhances ecological and comparative analyses by enabling reliable comparisons between populations, regions, or time periods. Angular angelshark biomorphometric data comprising sex, life stage, weight, and total length are depicted in Table 2 per sampling point.

**TABLE 2:** Angular angelshark (*Squatina guggenheim*) biomorphometric data [sex, life stage, total weight (kg), and total length (TL)] per sampling site in Rio de Janeiro, Southeastern Brazil.

Sampling point	Sex	Life stage	N	Weight (kg)	TL (cm)
Copacabana	Male	Adult	1	4.53^a^	84.9^a^
Recreio dos Bandeirantes	Female	Adult	5	5.22 ± 0.43	84.85 ± 3.32
		Juvenile	22	17.99 ± 81.97	38.08 ± 9.35
	Male	Adult	2	2.65 ± 3.03	62.75 ± 32.17
		Juvenile	13	0.56 ± 0.27	41.18 ± 5.38

^a^ One individual only.

### Metal and metalloid concentrations in angular angelshark meat

Metal and metalloid concentrations determined in angular angelshark muscle are depicted in Table 3, compared to concentrations determined in other Squatinidae family members and other benthic elasmobranch species. When necessary, reported data were converted to wet weight (w.w.) using 78% water content (Synnes et al., 2007; Barragán-Méndez et al., 2018).

**TABLE 3:** Metal and metalloid concentrations determined in the meat of angular angelshark (*Squatina guggenheim*) sampled in Rio de Janeiro, Southeastern Brazil, compared to concentrations determined in other Squatinidae family members and other benthic elasmobranch species. Units are all given as mg kg^−1^ wet weight.

Reference	Species	Sampling region	N	Element (mg kg^−1^ wet weight)														
				Ag	As	Cd	Co	Cu	Fe	MeHg	Mn	Pb	Rb	Se	THg	Ti	V	Zn
This study	*Squatina guggenheim*	Rio de Janeiro, Brazil	43	0.004^a^	4.42 ± 5.25	0.01 ± 0.001	0.02 ± 0.05	0.46 ± 0.78	6.25 ± 4.25	0.10 ± 0.12	0.19 ± 0.08	0.12^a^	0.74 ± 0.20	0.45 ± 0.31	0.11 ± 0.13	1.30 ± 0.44	0.02 ± 0.01	2.93 ± 0.83
Storelli et al. (2005)	*Scyliorhinus canicula*	Adriatic Sea, Italy	12	—	7.88 ± 2.92	—	—	—	—	1.01 ± 0.58	—	—	—	—	1.1 ± 0.62	—	—	—
Adel et al. (2018)	*Chiloscyllium arabicum*	Persian Gulf, Iran	40	—	—	0.04 ± 0.02	—	0.74 ± 0.23	—	—	—	0.08 ± 0.03	—	—	0.03 ± 0.03	—	—	2.40 ± 0.63
Lopes et al. (2019)	*Narcine brasiliensis*	Espírito Santo, Brazil	4	—	92.67 ± 50.12	0.089 ± 0.095	0.013 ± 0.007	0.47 ± 0.167	14.25 ± 9.475	—	0.14 ± 0.052	0.03 ± 0.026	—	1.083 ± 0.623	0.690 ± 0.116	—	0.195 ± 0.041	12.66 ± 2.717
Marques et al. (2021)	*Scyliorhinus canicula*	Atlantic Ocean, Portuguese fishers	74	—	—	—	—	0.352	1.06 ± 0.79	—	0.088 ± 0.018	0.143	—	0.58 ± 0.17	—	—	—	4.93 ± 1.04
Martins et al. (2021)	*Squatina guggenheim*	São Paulo, Brazil	9	—	—	0.02–0.05	—	0.01–0.47	5.44–12.8	—	—	—	0.004–0.12	—	—	—	—	—
Turan et al. (2021)	*Squatina aculeata*	Mediterranean Sea, Turkey	1	—	7.54 ± 0.65	—	—	0.01 ± 0.008	26 ± 8.25	—	0.035 ± 0.01	—	—	—	—	—	—	10.86 ± 1.73
Wosnick et al. (2021)	*Ginglymostoma cirratum*	Maranhão, Brazil	28	—	—	0.33	—	—	—	—	—	0.28	—	6.90	24.83	—	—	—
Shipley et al. (2021)	*Ginglymostoma cirratum*	Bahamas, Caribe	5	0.21 ± 0.21	6.56 ± 6.84	0.46 ± 0.53	0.02 ± 0.04	11.22 ± 7.72	—	—	1.12 ± 0.54	0.19 ± 0.09	—	—	15.80 ± 5.06	—	—	154.00 ± 50.02
Storelli et al. (2022)	*Tetronarce nobiliana*	Mediterranean Sea, Italy	27	—	—	—	—	—	—	1.10 ± 0.04	—	—	—	0.50 ± 0.03	1.22 ± 0.05	—	—	—
	*Torpedo*		29	—	—	—	—	—	—	0.74 ± 0.04	—	—	—	0.37 ± 0.02	0.87 ± 0.04	—	—	—
	*Torpedo marmorata*		32	—	—	—	—	—	—	1.03 ± 0.03	—	—	—	0.48 ± 0.03	1.14 ± 0.04	—	—	—
Higueruelo et al. (2024)	*Scyliorhinus canicula*	Barcelona, Spain	60	—	29.1 ± 8.2	0.002 ± 0.001	—	0.63 ± 0.115	—	—	—	0.008 ± 0.004	—	—	1.7 ± 0.62	—	—	18.5 ± 3.87
Marques et al. (2025)	*Ginglymostoma cirratum*	Maranhão, Brazil	10 (F)	—	23.1 ± 14.0	—	—	—	—	—	—	—	1.16 ± 0.15	0.32 ± 0.09	2.03 ± 0.69	2.03 ± 0.15	—	—
			4 (M)	—	3.98 ± 2.61	—	—	—	—	—	—	—	1.12 ± 0.25	0.52 ± 0.02	2.85 ± 2.08	2.08 ± 0.17	—	—

^a^ Only one result above the LOQ.

The present study is the first to report concentrations of Ag, Co, MeHg, total mercury (THg), Pb, Se, and Ti in the muscle of angular angelsharks. Cadmium concentrations were lower than those reported by Martins et al. (2021) for the same species captured off the coast of São Paulo, also in southeastern Brazil (0.06 mg kg^−1^ w.w.), while Rb levels were higher (0.12 mg kg^−1^ w.w.). Turan et al. (2021) detected higher As (7.54 ± 0.65 mg kg^−1^ w.w.) and Zn (10.86 ± 1.73 mg kg^−1^ w.w.) levels in the sawback angelshark (*Squatina aculeata*) from the Mediterranean than those determined herein (4.42 ± 5.25 mg kg^−1^ w.w. and 2.93 ± 0.83 mg kg^−1^ w.w., respectively).

Concerning benthic sharks, THg concentrations determined herein (0.11 ± 0.13 mg kg^−1^ w.w.) were considerably lower than those reported by Wosnick et al. (2021) in Atlantic nurse shark (*Ginglymostoma cirratum*) from the state of Maranhão, northeastern Brazil (24.83 mg kg^−1^ w.w.), by Marques et al. (2025) from the same region (2.85 mg kg^−1^ w.w.), and by Shipley et al. (2021) in the Bahamas (15.80 ± 5.06 mg kg^−1^ w.w.). Concerning Pb, levels detected herein were higher in *S. guggenheim* than those recorded by Lopes et al., (2019) for lesser numbfish (*Narcine brasiliensis*) in the state of Espírito Santo, southeastern Brazil (0.03 ± 0.026 mg kg^−1^ w.w.), and by Adel et al. (2018) for the Arabian carpetshark (*Chiloscyllium arabicum*) in the Persian Gulf (0.08 ± 0.03 mg kg^−1^ w.w.). Finally, Se levels were similar to those reported by Storelli et al. (2022) for Torpedinidae rays captured in the Italian sea (0.50 ± 0.03 mg kg^−1^ w.w. *Tetronarce nobiliana*; 0.37 ± 0.02 mg kg^−1^ w.w. *Torpedo torpeto* and 0.48 ± 0.03 mg kg^−1^ w.w. *Torpedo marmorata*).

The As concentrations observed in angular angelsharks (4.42 ± 5.25 mg kg^−1^ w.w.) were lower than those reported for *Scyliorhinus canicula* in the Adriatic Sea (7.88 ± 2.92 mg kg^−1^ w.w.; Storelli et al., 2005) and along the Spanish coast (29.1 ± 8.2 mg kg^−1^ w.w.; Higueruelo et al., 2024). Similarly, THg and MeHg levels in angular angelsharks (0.11 ± 0.13 and 0.10 ± 0.12 mg kg^−1^ w.w., respectively) were approximately 10 times lower than those reported for *S. canicula* from the Adriatic Sea (1.1 ± 0.62 and 1.01 ± 0.58 mg kg^−1^ w.w.; Storelli et al., 2005) and also lower than the THg values recorded in Spain (1.7 ± 0.62 mg kg^−1^ w.w.; Higueruelo et al., 2024). In contrast, Pb concentrations in angular angelsharks (0.12 mg kg^−1^ w.w.) were higher than those reported by Higueruelo et al. (2024) for *Scyliorhinus canicula* (0.008 ± 0.004 mg kg^−1^ w.w.) and similar to the values observed by Marques et al. (2021) in the Atlantic Sea (0.143 mg kg^−1^ w.w.).

Some less studied elements categorized as technology-critical elements, such as Rb and Ti, were lower in angular angelsharks than in nurse sharks (*G. cirratum*) from the state of Maranhão (1.16 ± 0.15 and 2.03 ± 0.15 mg kg^−1^ w.w., respectively) in northeastern Brazil (Marques et al., 2025), while Co values were similar to those reported by Lopes et al., (2019) for the lesser numbfish from the state of Espírito Santo, southeastern Brazil.

Regarding essential elements, Fe levels observed in the present study were lower than those reported by Lopes et al., (2019) for lesser numbfish (14.25 ± 9.475 mg kg^−1^ w.w.) from Brazil and for *S. aculeata* from the Mediterranean (26 ± 8.25 mg kg^−1^ w.w.) as reported by Turan et al. (2021), similar to those reported by Martins et al. (2021) for *S. guggenheim* in São Paulo (5.44–12.8 mg kg^−1^ w.w.) and higher than those recorded by Marques et al. (2021) for *Scyliorhinus ocellus* in the Atlantic (1.06 ± 0.79 mg kg^−1^ w.w.). Values for both Mn and Cu were similar to those described by Lopes et al., (2019) for lesser numbfish (0.14 ± 0.052 and 0.47 ± 0.167 mg kg^−1^ w.w., respectively) and lower than those reported by Shipley et al. (2021) for nurse sharks from the Bahamas (1.12 ± 0.54 and 11.22 ± 7.72 mg kg^−1^ w.w., respectively). Finally, Se concentrations in the present study were similar to those recorded by several other studies conducted worldwide, *i.e.*, *S. canicula* (Marques et al., 2021), *T. nobiliana*, *T. torped*, *T. marmorata* (Storelli et al., 2022), and nurse sharks from Maranhão (Marques et al., 2025) and lower than those in lesser numbfish (Lopes et al., 2019).

### Comparison to maximum permissible metal and metalloid limits

Consumption of contaminated animals may expose humans to cumulative toxic effects, potentially impairing physiological systems and increasing susceptibility to chronic diseases associated with metal and metalloid toxicity (Azmi et al., 2019; Ju et al., 2021; Souza-Araujo et al., 2021; Storelli et al., 2022).

In Brazil, metal and metalloid concentration limits are established by the Brazilian National Health Surveillance Agency (ANVISA) (República Federativa do Brasil, 1999), although limits have only been set for As (1.0 mg kg^−1^), Cd (0.05 mg kg^−1^), total Hg (1.0 mg kg^−1^), and Pb (0.3 mg kg^−1^) so far. It is important to note, however, that the Brazilian legislation presents challenges in interpretation and enforcement with respect to As. According to RDC No. 722 (01/07/2022), which is currently in force, the limit applies to total As, and speciation analyses to determine inorganic As contents in foodstuffs is only required if the total concentration exceeds 1 mg kg^−1^ *w.w.*, with compliance being verified using methodologies quantifying total As. The Ministry of Agriculture, Livestock and Food Supply (MAPA), however, adopts a different interpretation, considering the limit of 1.0 mg kg^−1^ to apply specifically to inorganic As, the toxicologically relevant form. This discrepancy between regulatory agencies has created ambiguity regarding compliance and enforcement for this element.

Several potentially toxic elements, such as Ni and V, however, have not been regulated so far, along with technologically critical elements of emerging concern, such as Pt, Rb, and Ti. In turn, the Food and Agriculture Organization of the United Nations (FAO) has established maximum metal and metalloid limits in foodstuffs only for Pb, at 0.3 mg kg^−1^, and for MeHg, at 1.0 mg kg^−1^ (FAO/WHO, 1995).

Cadmium, Hg, MeHg, and Pb concentrations in angular angelshark muscle (0.005 ± 0.001 mg kg^−1^, 0.12 ± 0.13 mg kg^−1^, 0.10 ± 0.12 mg kg^−1^, and 0.12 mg kg^−1^, respectively, the latter with only one sample above the LOQ) were all within legal Brazilian guidelines limits. Mean As, on the other hand, was approximately 4-fold higher (4.42 ± 5.25 mg kg^−1^) than the established ANVISA limit of 1.0 mg kg^−1^. When using the value determined in the most contaminated specimen, a much higher value of 25-fold above the limit (25.9 mg kg^−1^ w.w.) was observed. Although As is naturally found in the lithosphere, hydrosphere, and atmosphere, it has been reported worldwide in water (both surface and groundwater), soil, and foodstuffs, mainly rice at high concentrations (Medunić et al., 2020; Upadhyay et al., 2019). Exposure to this element may result in skin changes, including keratosis and hyperpigmentation, and different types of cancer, both cutaneous and in internal organs (Shahid et al., 2018).

### Interelemental correlations in angular angelshark

Interelemental correlations between metals and metalloids may suggest common elemental origins or similar biochemical processes (Norwood et al., 2003). The Spearman correlations between the determined metals and metalloids and TL, for both male and female angular angelsharks, considering both adults and juveniles and separated by sex and age class are depicted in Table 4. Only significant (p < 0.05) strong (r > 0.70) and very strong (r > 0.90) associations are reported.

**TABLE 4:** Significantly strong and very strong inter-element correlations between biomorphometric variables and metals and metalloids, and between the determined elements in angular angelshark (*Squatina guggenheim*) meat. Potentially protective essential element effects against toxic elements are marked in bold.

Associated variable		*p* value	Correlation coefficient	Correlation strength	Molar ratio	Group
Biometric parameters × metals	TL × Se	0.0458	0.73	Strong	—	Grouping all samples
	TL × Hg	0.0013	0.89	Strong	—	
	TL × MeHg	0.0013	0.88	Strong	—	
Metals × metals	Se × Ti	0.0589	0.70	Strong	0.21:1	Grouping all samples
	Se × As	0.0214	0.80	Strong	0.1:1	
	As × Hg	0.0020	0.89	Strong	107.58:1	
	**Se × Hg**	**0.0123**	**0.95**	**Very strong**	**10.39:1**	
	**Se × MeHg**	**0.0123**	**0.92**	**Very strong**	**11.43:1**	
	As × MeHg	0.0020	0.91	Very strong	118.34:1	
	As1% × Hg	0.0020	0.90	Very strong	0.97:1	
	Fe × Cu	0.0154	0.85	Strong	35.6:1	Only female
	Se × Ti	0.0167	0.94	Very strong	0.24:1	
	Fe × Co	0.0238	0.71	Strong	149.83:1	Only male
	**Cu × Hg**	**0.0333**	**0.85**	**Strong**	**37.25:1**	Only juvenile
	**Cu × MeHg**	**0.0333**	**0.85**	**Strong**	**37.25:1**	
	**Zn × Hg**	**0.0333**	**0.89**	**Strong**	**178.56:1**	
	**Zn × MeHg**	**0.0333**	**0.86**	**Strong**	**178.56:1**	
	Se × Ti	0.0167	1.00	Very strong	0.14:1	
	**Zn × Rb**	**0.0218**	**0.81**	**Strong**	**4.69:1**	Only adult

Concerning biometric variable associations, weight was significantly and strongly associated with total length, both when sexes (r = 0.87) were grouped and when female (r = 0.84) and male sharks (r = 0.94) were considered separately, as expected, since direct associations between these variables are common in fish and typically reflect biomass accumulation as the organism grows or reaches maturity (Silva et al., 2007). Total length, in turn, was positively correlated with both Hg and Se, explained by Hg bioaccumulation over time seemingly accompanied by a corresponding increase in Se concentrations. This association is commonly reported in fish and indicates a protective role of Se against Hg and its methylated form, which will be discussed ahead (Censi et al., 2006; Burger et al., 2012; Delshad et al., 2012; Julio et al., 2022).

The combined effects of toxic elements can increase or decrease damage to health. Synergistic effects between metals can result in both higher and lower toxicity, depending on each of their concentrations and properties (Goyer, 1997; Cedergreen, 2014). Correlations between essential elements indicate interconnected metabolic processes, while correlations among toxic elements may reflect a common environmental source or similar detoxification pathways (Land et al., 2018; Hauser-Davis 2020). Associations between essential and toxic elements, in turn, may indicate a potential role of the former in mitigating the harmful effects caused by the latter (Land et al., 2018). In this sense, when the molar concentration of an essential metal exceeds that of a toxic metal, the essential element may function as a protective agent, counteracting or buffering the harmful impacts of the toxicant helping reduce toxicity in exposed organisms (Cuvin-Aralar and Furness, 1991; Sørmo et al., 2011). A molar ratio between essential and toxic elements greater than 1 is indicative of protective effects.

Although several strong and very strong correlations were noted between many metals in angular angelshark meat, potentially protective effects, *i.e*., excess essential element concentrations compared to toxic elements, were noted only for seven of them (depicted in bold in Table 4), considering both total Hg and MeHg contents, six of them concerning Hg toxicity and one concerning Rb toxicity.

The Se × Hg relationship is a well-established protective interaction in fish, including elasmobranchs (Kaneko and Ralston, 2007; Ralston and Raymond, 2010; Wosnick et al., 2021; Storelli et al., 2022). A molar ratio of 10.39 to 1 was observed concerning this association in angular angelshark meat, increasing to 11.43 to 1 when considering Hg in its methylated form, indicating excess Se concentrations compared to Hg and, thus, a protective effect. Selenium exhibits a strong affinity for Hg due to its electropositive nature and its ability to form stable covalent bonds, leading to the formation of biologically inert compounds, such as mercury selenide (HgSe). These complexes significantly reduce the bioavailability and toxicity of both inorganic Hg and MeHg in aquatic organisms and humans (Burger et al., 2012; Scheuhammer et al., 2015). This interaction may occur both intra- and extracellularly, with Hg–Se complexes being directed toward organelles or sequestered in electron-dense granules, effectively isolating mercury and limiting its toxic action (Chen et al., 2006). The strong affinity of both Se and Hg for sulfhydryl (−SH) groups, which is common in cysteine-rich biomolecules, further promotes the formation of these complexes, particularly with selenoproteins. These proteins play key antioxidant roles and contribute to the protection of vital cellular structures from Hg-induced oxidative damage (Sugiura et al., 1978; Chen et al., 2006).

The other observed correlations regarding potentially protective effects were also noted for Hg and MeHg, in particular their associations with Cu and Zn. Essential metals, such as Cu and Zn, are known to stimulate the production of regulatory and defense proteins, such as metallothioneins, which can bind to Hg (and other elements), facilitating its retention and reducing its reactivity within exposed organisms (Ziller and Fraissinet-Tachet, 2018; Wang et al., 2021). Additionally, some essential elements, such as Zn, may aid in mitigating harmful Hg effects by competing with this toxic element for binding sites (Szunyogh et al., 2015). In this sense, Hg is known to interfere with the function of proteins and enzymes that rely on essential metals such as Cu, Fe, and Zn to maintain their structure and activity (Balali-Mood et al., 2021), displacing these critical elements from their protein-binding sites, disrupting vital functions such as oxygen transport, cellular protection against oxidative damage, and energy production, and also inducing structural changes that inactivate these molecules and impair overall cellular function (Huang et al., 2006; Tandogan and Ulusu, 2006). The molar ratios noted for Cu and Zn, however, were higher (37.25 and 178.56 to 1, respectively), indicating a more stable excess essential element molar ratio than that of Hg and MeHg.

Interestingly, the consistent molar ratios observed for both MeHg and Hg in association with Cu, as well as between both Hg forms and Zn, suggest a potential underlying stoichiometric relationship or shared binding mechanism between these Hg species and these transition metals, strongly implying a conserved ligand–metal interaction. This could indicate that, despite their distinct chemical forms, both MeHg and inorganic Hg may participate in similar chelation processes, for example, utilizing similar (−SH) or nitrogen-containing ligands within the environmental matrix, which then coordinate with Cu and Zn, or coordination complexes with these divalent cations, potentially driven by shared electron donor groups or geometric constraints within the binding sites (Sauliutė and Svecevičius, 2015). Such stoichiometric constancy might reflect specific thermodynamic preferences, *i.e*., the most thermodynamically stable configurations for Hg–metal complexes under the given conditions or similar kinetic pathways governing Hg–metal associations in the system.

Finally, a positive correlation between Zn and Rb was observed in adult individuals. Studies in fish are scarce, but Yokoi et al. (1996) demonstrated that Rb deficiency in rats can affect the status of several minerals, including decreased Zn levels in plasma and testicular tissue, highlighting a potential regulatory interaction. More investigations, in this sense, in fish, however, are required to corroborate or refute this hypothesis.

### Selenium health benefit

The Se-HBV was developed to assess the health benefits of Se in foodstuffs considering both dietary Se intake and exposure to MeHg simultaneously (Kaneko and Ralston, 2007; Ralston, 2008; Ralston et al., 2016). The original formula was, however, later revised as it could produce disproportionate results when Hg concentrations were significantly lower than Se levels (Ralston et al., 2016). The modified equation, known as HBV_Se_, more accurately reflects the excess concentration of Se relative to that of MeHg in food, offering a more reliable estimate of Se’s protective potential. The values of both the original and modified versions calculated herein are presented in Table 5 and compared to values reported in studies on other elasmobranch species.

**TABLE 5:** Comparisons between original and Selenium Health Benefit and Modified Selenium Health Benefit value (Se-HBV and HBV_SE_, respectively) determined in angular angelshark (*Squatina guggenheim*) meat in the present study compared to other shark species worldwide. These indices are unitless.

Species	Depth range (m)	Sampling point	Se-HBV	HBV_SE_	References
*Squatina guggenheim*	10–80	Rio de Janeiro, Brazil	1.53 ± 1.36	0.285 ± 0.282	Present study
*Isurus oxyrinchus*	0–888	Honolulu, Hawaii, United States	−11.1	—	Kaneko & Ralston, (2007)
*Alopias vulpinus*	0–650		2.5	—	
*Isurus oxyrinchus*	0–888	Honolulu, Hawaii, United States	—	−16.44 ± 8.57	Ralston et al. (2019)
*Alopius vulpinus*	0–650		—	2.67 ± 2.04	
*Carcharhinus falciformis*	18–500	Baja California Sur, Mexico	4.9	—	Terrazas—López et al. (2019)
*Sphyrna zygaena*	1–200		17.7	—	
*Prionace glauca*	0–1,000	Mediterranean Sea, Italy	—	−1.36	Storelli et al. (2022)
*Squalus acanthias*	0–1978		—	−2.6	
*Squalus blainville*	15–1,500		—	2.69	
*Mustelus mustelus*	5–438		—	1.96	
*Mustelus asterias*	0–199		—	2.26	
*Scyliorhinus canicula*	0–800		—	3.41	
*Carcharhinus falciformes*	18–500	Trinidad Tobago	—	93.41 ± 126.27	García Barcia et al. (2023)
*Carcharhinus altimus*	0–810		—	3.67	
*Mustelus canis*	0–808		—	−7.41 ± 20.28	
*Mustelus norrisi*	0–260		—	1.35 ± 2.01	
*Mustelus higmani*	0–130		—	4.92 ± 3.18	
*Rhizoprionodon lalandii*	3–149		—	4.96	
*Rhizoprionodon porosus*	0–500		—	−0.98	
*Rhizoprionodon terraenovae*	0–280		—	5.09 ± 3.51	
*Sphyrna lewinii*	0–1,043		—	−1.39 ± 7.43	
*Sphyrna mokarran*	0–300		—	−12.34 ± 22.56	
*Mustelus californicus*	12–67	Baixa California Sur, Mexico	—	0.35	Pantoja-Echevarría et al. (2024)
*Sphyrna zygaena*	1–200		—	1.61	
*Isurus oxyrinchus*	0–888		—	0.051	
*Mustelus henlei*	1–266		—	0.2	

The modified HBVSeSe, despite yielding a much lower numerical value (0.29 ± 0.29) than the original (1.53 ± 1.36), offers a more conservative and biologically meaningful assessment of whether Se levels are sufficient to mitigate MeHg-associated risk, providing the most reliable basis for risk–benefit assessment.

Negative Se-HBV and HBVSe values indicate that MeHg concentrations are in excess of the protective amount of Se present in the analyzed matrix, suggesting that there is insufficient Se to counteract potential Hg toxicity. In contrast, positive values suggest that Se is present in excess relative to MeHg, providing a potential protective effect against mercury-related risks. Herein, the overall mean values for both Se-HBV and HBVSe reported for angular angelshark meat were positive, indicating a protective effect of Se against Hg toxicity concerning the consumption of angular angelshark meat. The HBVSe value calculated for this species was only surpassed by that reported for the shortfin mako (*Isurus oxyrinchus*) by Pantoja-Echevarría et al. (2024). Similarly, the Se-HBV value in the present study exceeded only that reported for the shortfin mako (Kaneko and Ralston, 2007) while remaining lower than those observed for the smooth hammerhead (*S. zygaena*) (Terrazas-López et al., 2019) and common thresher (*Alopias vulpinus*) (Kaneko and Ralston, 2007). The high values observed herein may indicate an Se-rich food source for angular angelsharks in southeastern Brazil, or, conversely, exposure to low Hg levels, along with efficient pathways for excreting or otherwise metabolizing Hg that contribute to a favorable balance between these elements. However, even though the values indicate protective Se effects, the presence of other toxic and potentially harmful elements and their human health risk calculations, discussed in the next sections, still indicate high risks associated with the consumption of angular angelshark meat.

### Estimated daily intakes (EDIs) and estimated weekly intakes (EWIs)

**FIGURE 2:**
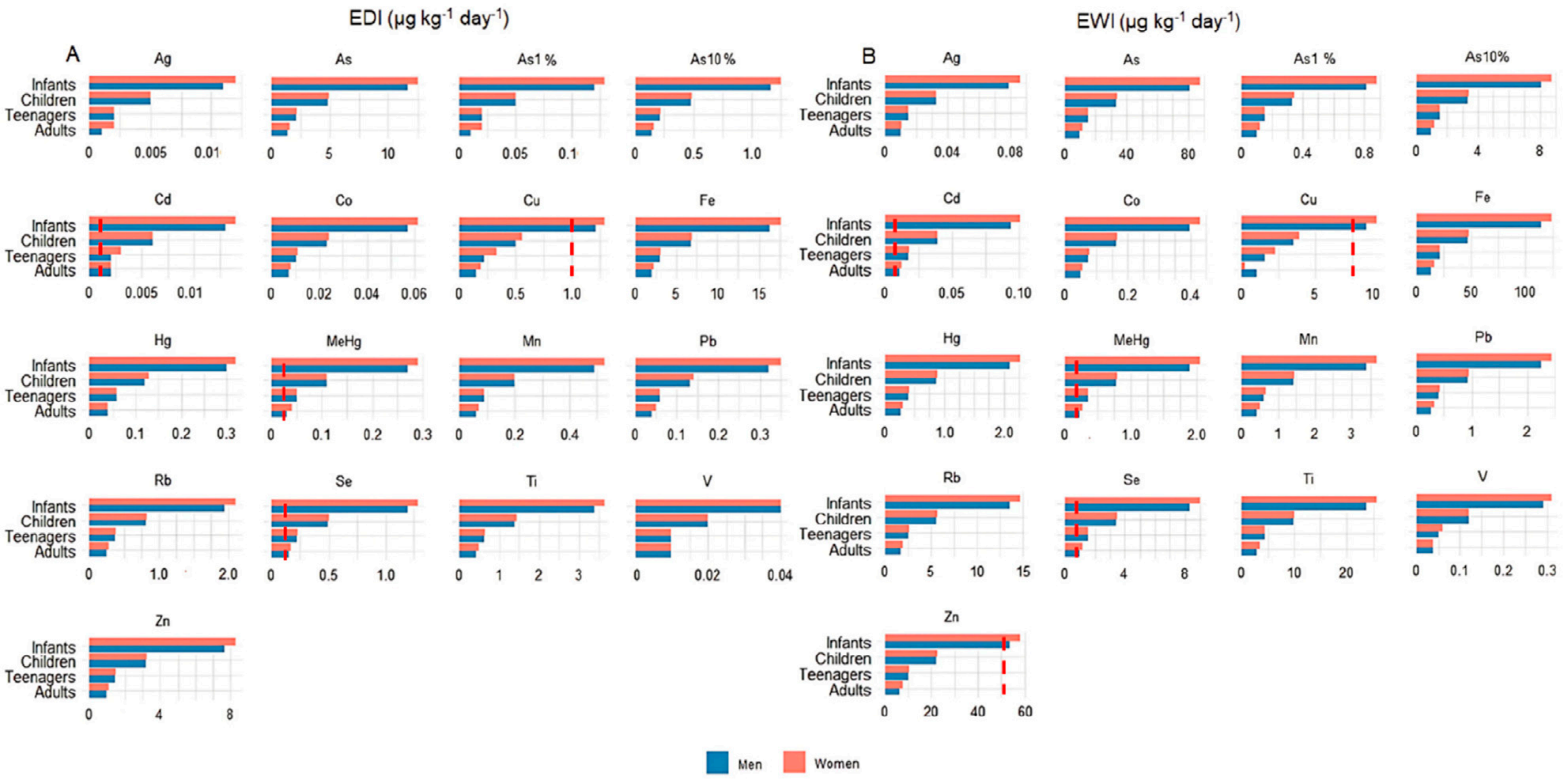
Estimated daily intake (EDI) **(A)** and estimated weekly intake (EWI) **(B)** per human age class and sex for metals and metalloids determined in the meat of angular angelsharks (*Squatina guggenheim*) sampled from Rio de Janeiro, Southeastern Brazil. The hatched bar (in red) corresponds to established EDI and EWI limits.

Brazil lacks daily and weekly guidelines concerning the concentration limits of metals and metalloids in food. Thus, the Estimated Daily Intake (EDI) and Estimated Weekly Intake (EWI) values calculated for angular angelshark meat consumption were compared with those in the international standards established by the FAO of the United Nations and the EU (Figure 2). It is important to note that few studies assess different human age groups and body weights when evaluating the health risks associated with elasmobranch meat consumption. Most research, in fact, focuses primarily on adults, leaving significant gaps in risk assessments specifically targeting children, which is the most vulnerable group due to the developing immune systems and lower body weights. Although some studies distinguish between male and female consumers, they often apply standardized body weights, typically 70 kg for men, 60 kg for women, and 16 kg for children, when included, (Barone et al., 2021; Yacoubi et al., 2023), which may not be the reality of different countries. Moreover, authors use considerably variable Fish Ingestion Rate (FIR) values across different regions, which complicates comparisons and limits more in-depth risk analyses.

The EDI and EWI for Cd and MeHg exceeded established guidelines for all evaluated parameters and age groups, while the EDI for Se was above the limits set by the European Commission in the worst case scenario for all age groups and for the average concentration in teenagers, children, and infants. Copper and Zn were above the limits in the worst case scenario, with the concentration calculated using the most contaminated specimen, for children and infants, and in the average concentration scenario for infants.

Concerning studies on the risks of consuming elasmobranch meat, Barone et al. (2021) evaluated the EDI for Se in three ray species (*Leucoraja circularis*, *Dipturus oxyrinchus*, and *Leucoraja fullonica*) sampled from the Apulia region of southern Italy, applied using an FIR of 37.2 g day^−1^ and a body weight of 26.2 kg, which according to that study corresponds to children aged 4–8 years, and in the present study would be the equivalent to children aged 6 years. Under these conditions, the highest EDI value for Se was noted in *L. circularis*, at 0.67 μg kg^−1^ bw day^−1^, higher than the limit established by EFSA (in 4–6 years, 0.09 μg kg^−1^ bw day^−1^ and in 7–10 years, 0.13 μg kg^−1^ bw day^−1^). When compared with the children in the present study, the value reported by Barone et al. (2021) was higher than 0.49 μg kg^−1^ bw day^−1^. Despite this difference, it is important to note that the FIR used in the present assessment was 24.38 g day^−1^, while that used by Barone et al. (2021) was 37.2 g day^−1^. In turn, Yacoubi et al. (2023) reported the EDI of 12 elements, of which nine (As, Cd, Cu, Fe, Hg, Mn, Pb, V, and Zn) were also evaluated in the present study, for children (16 kg), adult women (71.7 kg), and adult men (77.3 kg) applying an FIR of 31.51 g day^−1^, assessed concerning six shark species (*Carcharhinus dussumieri*, *Carcharhinus sorrah*, *Chiloscyllium arabicum*, *Gymnura poecilura*, *Sphyrna lewini*, and *Sphyrna mokarran*) collected in the Arabian Gulf. The highest EDI values and, consequently, the highest risk were observed for 6-year-old children. Da Silva et al. (2025) evaluated the EDI in *Dasyatis* spp., Caribbean sharpnose shark (*Rhizoprionodon porosus*), and *Z. brevirostris* from Farol de Sao Tomé, along with the state of Rio de Janeiro, applying an FIR of 26.7 g day^−1^ and weights for adult men and women of 67 and 60 kg, respectively. The highest EDI value for As was observed in *Zapteryx brevirostris*, with values above 3,000 μg kg^−1^ bw day^−1^, while the highest EDI value for Se was observed in *Caribbean sharpnose shark*, with values of 370 μg kg^−1^ bw day^−1^, both of which are very high when compared to those in the present study.

The EDI for As, Cd, Hg, Zn, and V for children and adults of both sexes reported in other studies were lower than those reported herein, while the remaining elements varied according to the age group. These differences can be attributed, in part, to variations in the parameters adopted by the authors, such as the weight considered for each age group and the FIR, which can vary significantly between populations and studies. For example, the FIR used by Yacoubi et al. (2023) was higher than that applied in the present study, directly influencing exposure estimates. This highlights the need to consider such methodological differences when comparing human health risks associated with the consumption of elasmobranch meat.

All elements that exceeded the established limits for EDI also surpassed the corresponding EWI values. This outcome is expected since the EWI is derived directly from the EDI by multiplying by 7, reflecting cumulative exposure over the course of a week. Thus, any exceedance of the daily limit automatically translates into an exceedance of the weekly limit, reinforcing the potential health risks associated with regular consumption of the contaminated fish.

### Target hazard quotient (THQ)

**FIGURE 3:**
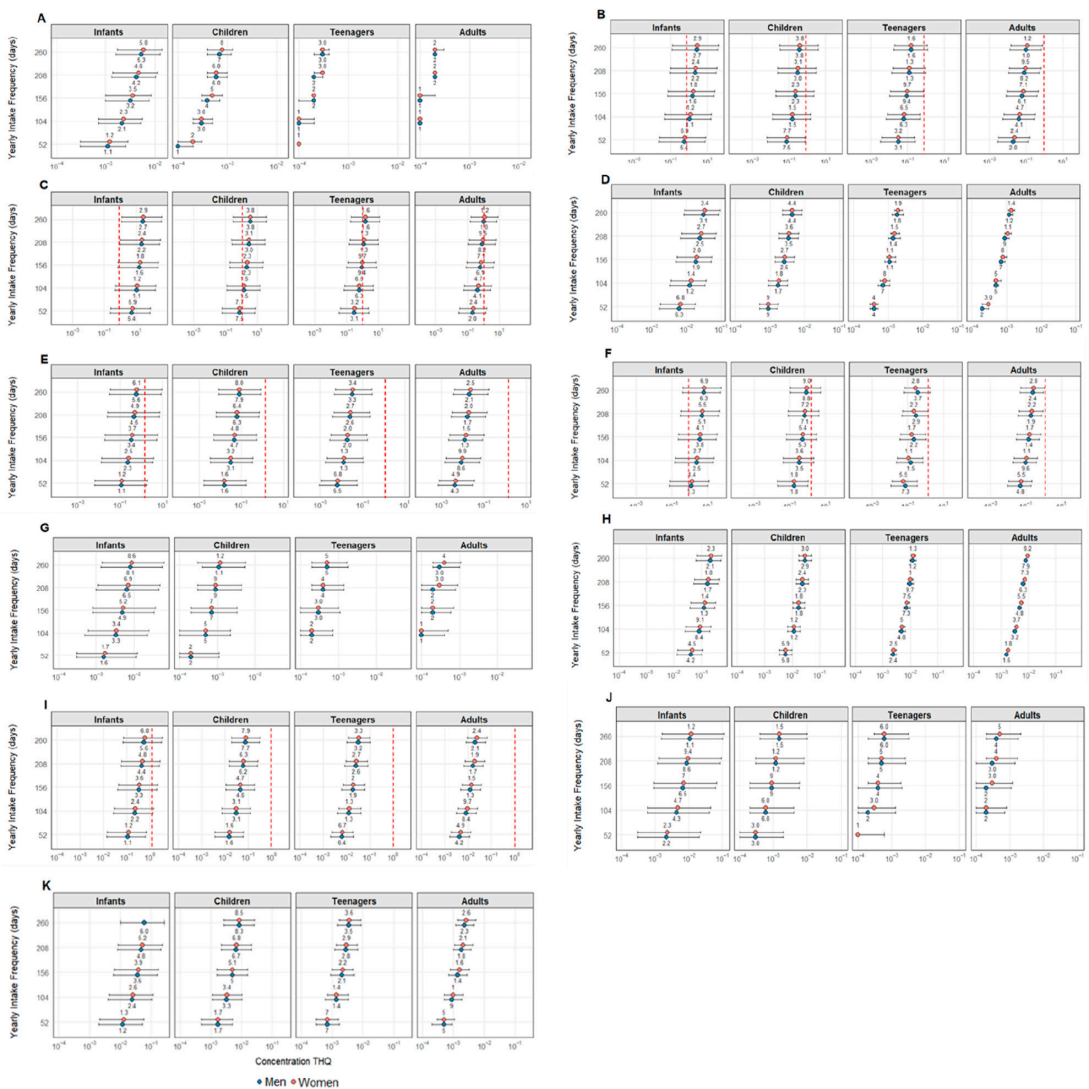
Target hazard quotient (THQ) results for **(A)** Ag; **(B)** As 1%; **(C)** As 10%; **(D)** Cd; **(E)** Cu; **(F)** MeHg; **(G)** Mn; **(H)** Pb; **(I)** Se; **(J)** V; and **(K)** Zn concerning angular angelshark (*Squatina guggenheim*) meat consumption. Data are presented considering a consumer ingestion frequency (EFR), expressed as days per year or times per week: 1× week = 52 days; 2× week = 104 days; 3× week = 156 days; 4× week = 208 days; and 5× week = 260 days. The “Sex” variable refers to consumer gender (boys and men, girls and women), and “Age Group” comprises infants, children, adolescents, or adults. The “Value” column represents different exposure scenarios (mean, maximum, and minimum), calculated using the average, maximum, and minimum concentrations for the determined elements in angular angelshark (*Squatina guggenheim*) muscle tissue. The maximum THQ value of 1 over which potential health risks are noted is indicated as a red vertical line in each graph.

The cumulative health risk caused by exposure to multiple elements should be considered when investigating human health risks from the consumption of contaminated foodstuffs as, even if a single element alone does not pose a health risk, their combined effects may lead to significant adverse health outcomes (Boldrocchi et al., 2019). In this sense, the target hazard quotient (THQ), as shown in Figure 3, was calculated for angular angelshark meat consumption frequencies ranging from 1 to 5 days per week. This index, however, could not be calculated for total As (only iAs), Co, Fe, Hg, Rb, and Ti due to the lack of established oral reference doses for these elements so far.

Only five elements (As 1%, As 10%, Cu, MeHg, and Se) exceeded the safety threshold, with THQ values over 1. Among them, As 10% showed the most critical results as, based on the most contaminated specimen, THQ > 1 was observed across all age groups, ingestion frequencies, and sexes. For As 1%, the maximum THQ exceeded 1 for infants consuming the species twice per week. Regarding As 10%, even at the lowest ingestion frequency (once per week), the mean THQ exceeded 1 for infants only. At higher frequencies, the risk increased significantly: in addition to mean values exceeding 1 for all age groups, minimum THQ values (based on the least contaminated specimen) also exceeded the threshold for infants of both sexes. As 10% was the element with the highest THQ values, with mean THQs reaching 41.5 for female infants. The maximum THQ for female infants exceeded 415 times the safety threshold, indicating an extreme potential health risk from consuming this species.

Compared to other assessments, considerably higher values were observed herein than values reported by Maciel et al. (2021), in which THQ for As 10% in the Caribbean sharpnose shark caught in Tamoios and Cabo Frio (both in Rio de Janeiro) was 0.81, when calculated using the mean concentration, and 1.50 based on the most contaminated specimen. These calculations, however, considered only adults weighing 70 kg, with an exposure duration (ED) of 76.3 years, an exposure frequency (EFR) of 365 days per year (*i.e.*, daily consumption), and an FIR of 24.74 g day^−1^. These values are considerably lower than those found in the present study.

In another study, Yacoubi et al. (2023) evaluated six species collected in the Arabian Gulf, considering an exposure duration of 75 years and an EFR of 365 days, categorizing consumers into three groups: women weighing 71.7 kg, men weighing 77.3 kg, and children weighing 16 kg. Their calculations were based on total arsenic content, but using the limit established for inorganic As (1 × 10^−4^), with the following THQ results: whitecheek shark (*C. dussumieri*): children 82.3, women 18.3, and men 17.0; spottail shark (*C. sorrah*): children 94, women 21, and men 19.5; Arabian carpetshark (*C. arabicum*): children 377, women 84.2, and men 78.1; longtail butterfly ray (*G. poecilura*): children 187, women 41.9, and men 38.8; scalloped hammerhead (*S. lewini*): children 64.4, women 14.3, and men 13.3; great hammerhead (*S. mokarran*): children 54.4, women 12.1, and men 11.3. Despite these high values, it is important to highlight that the calculations assumed an EFR of 365 days, *i.e.,* daily consumption throughout the entire year, unlike the present study, in which alarming results are noted even with lower consumption frequencies.

Another study that assessed THQ for As 10% in specimens collected in the northern region of Rio de Janeiro was conducted by Da Silva et al. (2025), who analyzed *Dasyatis* spp., Caribbean sharpnose shark, and shortnose guitarfish (*Z. brevirostris*) for two consumer groups: women weighing 60 kg and men weighing 67 kg, with an EFR of 52 days per year (weekly consumption) and an FIR of 26.79 g day^−1^. All species exceeded the safe limit. For *Dasyatis* spp., the THQ was approximately 12 in women and 10.5 in men, while Brazilian sharpnose sharks showed the lowest values, approximately 1.05 in men and 1.1 in women, and shortnose guitarfish reached 19 for women and 17 for men.

Methylmercury, even at the lowest intake, exceeded the limit in the average scenario for infants in the present study. The worst scenarios for all elements were for infant girls in the case of MeHg, due to their lower body sizes, reaching average and maximum values ​​of 6.87 and 59.40, respectively. Da Silva et al. (2025) reported the highest THQ for MeHg in adults from *Z. brevirostris* consumption of 14.9 for men and 16.0 for women, while the other two species assessed by these authors, *Dasyatis* spp. and Caribbean sharpnose shark, were well below the limit of 1, with values of less than 0.10.

Concerning Cu, only the maximum values for infants were exceeded at an angular angelshark consumption frequency of once a week. No other age group exceeded the limit for this element. In one study, Adel et al. (2018) assessed Arabian carpetsharks (*C. arabicum*) from Persian Gulf applying an FIR of 227 g, a body weight of 70 kg, an EFR of 7 days, 1 day per week (365 days year^−1^ and 52 days year^−1^, respectively), and an Ed of 70 years. The calculated THQ value for Cu regarding consumption once a week was 6.90 × 10^−4^, and for seven times a week, 4.81 × 10^−3^. In another study conducted on sharks sampled from the Persian Gulf, Heidarieh et al. (2025) assessed the grey sharpnose shark (*Rhizoprionodon oligolinx*) and milk shark (*Rhizoprionodon acutus*), calculating consumption risks for frequencies of once and seven times a week. The calculated Cu THQs were 6.76 × 10^−4^ and 4.7 × 10^−3^ for the former and 3.7 × 10^−4^ and 2.5 × 10^−3^ for the latter, none exceeding established guidelines.

However, Yacoubi et al. (2023) reported THQ values for Cu ranging from 1.19 × 10^−2^ (*S. lewini*) and 2.09 × 10^−2^ (*C. dussumieri*) in children; 2.65 × 10^−3^ and 4.67 × 10^−3^ in adult women; and 2.44 × 10^−3^ and 4.33 × 10^−3^ in adult men for the same species, collected at the fishing port of Jubail (located on the northern coast of Saudi Arabia in the Arabian Gulf). Jha et al. (2024) evaluated 11 shark species from the Arabian Sea and calculated THQ values only for 70 kg adults, reporting that none of the species exceeded the THQ limit of 1 for Cu, with the highest value reported for spadenose sharks (*Scoliodon laticaudus*) (4.47 × 10^−2^).

Pantoja-Echevarría et al. (2024) evaluated the THQ for Se in brown smoothhounds (*Mustelus henlei*), gray smoothhounds (*Mustelus californicus*), smooth hammerheads (*Sphyna zygaena*), and shortfin makos captured at the Bay of California in Mexico for children weighing 16 kg and adults weighing 70 kg. The findings were reported as 0.033, 0.005, 0.01, and 0.004 for adults and 0.006, 0.01, 0.02, and 0.01 for children, corresponding to brown smoothhounds, gray smoothhounds, smooth hammerheads, and shortfin makos, respectively, confirming that the lower the age group and, consecutively, the body weight, the greater the risk of shark meat consumption, even though none of the species exceeded the limit (THQ > 1).

Da Silva et al. (2025) analyzed the THQ for three elasmobranch species, reporting that only Caribbean sharpnose sharks exceeded the limit, with values slightly below 1.25 for both men and women (adults). Other age groups, however, were not evaluated.

### Hazard index (HI)

**FIGURE 4:**
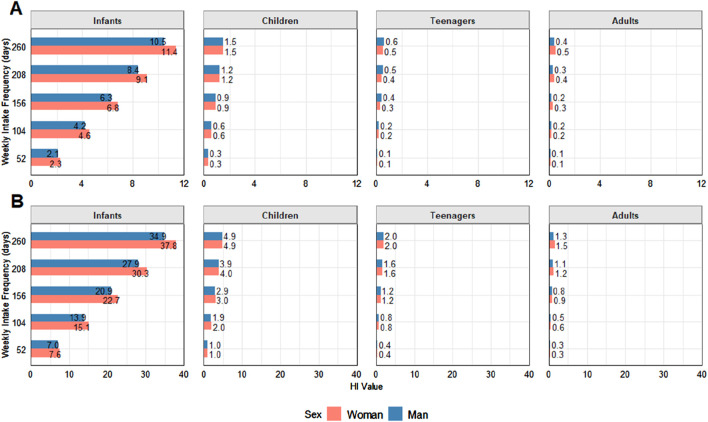
Hazard index (HI) results for each assessed age class at 1–5 weekly intake frequencies for both sexes. Concerning As, the As1% **(A)** and As10% **(B)** scenarios were used to estimate iAs contributions. Values above 1 indicate health risks.

Metal toxicity does not take place in isolation, with metal–metal interactions potentially aggravating human health risks, as exposure to multiple metals can result in synergistic and additive effects, where the combined toxicity is greater than the sum of the individual effects (Castilhos, 2005). In this sense, the HI is a valuable tool used to assess potential health risks caused by exposure to various contaminants. However, not all metals and metalloids evaluated herein could be included in the HI calculation as no RfD has been established for total As, Co, Fe, Hg, Rb, and Ti, resulting in no THQ values and, therefore, no inclusion in the HI sum of THQs, which likely led to the underestimated values. Thus, only inorganic As (1% and 10% scenarios, based on total As values), Cd, and Pb were used for the HI calculation. Values below 1.0 indicate no potential risk, values from 1 to 10 indicate potential risks, and HI values above 10 indicate a high probability of potential health risks (Yee, 2010). The calculated HI results for each assessed age class at 1–5 weekly intake frequencies for both sexes are depicted in Figure 4. For As, the 1% and 10% As scenarios were used to calculate the iAs contributions to this index to not overestimate HI values (Akter et al., 2005).

At the minimum frequency, calculated as once a week, maximum values in the As 10% scenario were above the limit for all age groups, while the means exceeded for both sexes in the As 1% and 10%. It is worth noting that HI values > 10 indicate a high probability of potential health risks (Yee, 2010), and at a frequency of 5 days per week, consumption values were above 10 for both male and female infants. In the worst possible average scenario at a frequency of 5 days per week, the calculated As 10% value was 273.8.

Although some studies concern human health risks regarding elasmobranch meat intake, none cover so many weight, age group, and intake frequency (THQ) categories as the present study.

According to Jha et al. (2024), of 11 elasmobranch species collected in India, only two (*C. sorrah* and *C. arabicum*) presented HI > 1, of 3.13 and 1.27, respectively. It is important to note, however, that those authors did not consider As, which may have underestimated the calculated HI values. Ju et al. (2021) evaluated the consumption of megamouth sharks (*Megachasma pelagios*) sampled in Taiwan, reporting HI values over 1 for adults, with higher risks noted for men (1.11) than for women (1.080), in contrast to the present study. In another study, blue sharks (*P. glauca*) and smooth hammerheads were assessed by Lara et al. (2022), where the HI was calculated by summing the THQ of Cd and Hg for three groups, namely men weighing 70 kg, women weighing 60 kg, and children weighing 16 kg, also categorizing the shark samples by sex. Most human age groups presented HI < 1, although children who consumed male blue sharks exhibited an HI of 1.32, indicating a high risk of exposure, similar to that observed herein for younger age groups. Pantoja-Echevarría et al. (2024) evaluated four species (*M. californicus*, *S. zygaena*, *I. oxyrinchus*, and *Mustelus henlei*), concerning adults weighing 70 kg, pregnant or lactating women weighing 60 kg, and children weighing 16 kg. The HI comprised the sum of Hg, Se, and in some cases Cd. THQs were lower than that for all species and human age groups. Yacoubi et al. (2023) evaluated six elasmobranch species (*Carcharhinus dussumieri*, *Carcharhinus sorrah*, *C. arabicum*, *G. poecilura*, *S. lewini*, and *S. mokarran*) from Saudi Arabia, Persian Gulf, calculating the HI for children, adult men, and adult women. The consumption of all shark species resulted in HI values over 10, for all age groups, ranging from 67.29 to 394.89 for children, 15.02 to 88.12 for women, and 13.93 to 81.74 for men, with the lowest value referring to *S. mokarran* and the highest, for *C. arabicum*, indicating extremely high consumption risks and a higher vulnerability for younger age groups, as noted herein.

### Carcinogenic risk and target cancer risk

It is important to note that several studies concerning elasmobranch meat consumption risks use the terms CR and TCR interchangeably, which they are not. The CR represents the probability of an individual developing cancer over a lifetime due to exposure to a single carcinogenic substance, calculated using the chronic daily intake and the chemical’s cancer slope factor, while TCR is the sum of individual CRs from all carcinogenic substances present, providing an overall estimate of lifetime cancer risk from combined exposures. Only two assessments concerning the CR have been conducted regarding elasmobranchs so far as most assessments are limited to TCR calculations only.

### Carcinogenic risk

The CR represents the probability of an individual developing cancer over his or her lifetime as a result of exposure to only one potentially carcinogenic agent, considering the average time of exposure to the carcinogen (Atc), EFR, ED, and the oral cancer slope factor for (for inorganic As, 1.5 mg kg^−1^ w.w. day^−1^) USEPA (2000). The acceptable lifetime carcinogenic risk limit as established by the USEPA is 10^−5^ (1 in 100,000 chances of developing cancer during a human lifetime). The CR was calculated, separating for both sexes and age groups using the average, maximum (most contaminated specimen), and minimum (least contaminated specimen) values, for As 1% and As 10% (Table 6).

**TABLE 6:** Hazard index (HI) results for each assessed age class at 1–5 weekly intake frequencies for infants, children, teenagers, and adults of both genders. The As 1% (A) and As 10% (B) scenarios were used to estimate inorganic As contributions. Values in bold depict carcinogenic health risks.

Sex	Age	CR				
		Total As	As 1%	As 10%	Cd	Pb
Male	Infants	1.73E+01	**1.73E−01**	**1.73E+00**	**1.34E−05**	**1.15E−03**
	Children	7.17E+00	**7.17E−02**	**7.17E−01**	5.52E−06	**4.75E−04**
	Teenagers	3.18E+00	**3.18E−02**	**3.18E−01**	2.45E−06	**2.11E−04**
	Adults	2.13E+00	**2.13E−02**	**2.13E−01**	1.64E−06	**1.41E−04**
Female	Infants	1.88E+01	**1.88E−01**	**1.88E+00**	**1.45E−05**	**1.24E−03**
	Children	7.30E+00	**7.30E−02**	**7.30E−01**	5.62E−06	**4.84E−04**
	Teenagers	3.30E+00	**3.30E−02**	**3.30E−01**	2.54E−06	**2.19E−04**
	Adults	2.46E+00	**2.46E−02**	**2.46E−01**	1.90E−06	**1.63E−04**

All CR values exceeded the limit (10^−5^) for all age groups concerning As and Pb, even when considering the least contaminated angular angelshark specimen. For Cd, only infants (both genders) were at risk.

In this sense, Pantoja-Echevarría et al. (2024) evaluated the CR for Cd, but not for As, while Yacoubi et al. (2023) evaluated As, Cd, Cr, Ni, and Pb in six species (*C. dussumieri*, *C. sorrah*, *C. arabicum*, *G. poecilura*, *S. lewini*, and *S. mokarran*) sampled from Jubail Port, located on the northern coast of Saudi Arabia, Persian Gulf, categorizing the data into three age classes, children (body weight of 16 kg), adult men (body weight of 77.3 kg), and adult women (body weight of 71.7 kg). When compared with As from the present study, children weighing 16 kg would correspond to ages 5 and 6 in Brazil UNIMED, (2021), so the data reported by Yacoubi et al. (2023) were compared herein to the child age group, excluding infants. In addition, those authors analyzed total As, and not its inorganic fraction, thus overestimating their results. All the ranges reported for the Persian Gulf were above the CR limit, with the highest values noted for children. All mean values for children in the present study were well below the values reported by Yacoubi et al. (2023). Concerning Cd and Pb, Yacoubi et al. (2023) also reported the greatest risks for children, with *C. arabicum* identified as the most concerning regarding Cd (2.70 × 10^−3^ for children, 6 × 10^−4^ for adult women, and 5.56 × 10^−4^ for adult men), while *S. lewini* presented the greatest risks concerning Pb (5.19 × 10^−4^ for children, 1.16 × 10^−4^ for adult women, and 1.07 × 10^−4^ for adult men). This contrasts with what was noted in the present study, where both male and female individuals presented CR–Cd values above the limit, differently from what was reported by both Yacoubi et al. (2023) and Pantoja-Echevarría et al. (2024), which only evaluated CR for Cd in *Mustelus californicus* collected in Baja California Sur, Mexico, for men weighing 70 kg (6.8 × 10^−5^) and women weighing 60 kg (9.8 × 10^−5^). Lead CR values were above the limit for all age groups and both sexes, but still lower than the values reported by Yacoubi et al. (2023) for *S. lewini*.

### Target cancer risk

**FIGURE 5:**
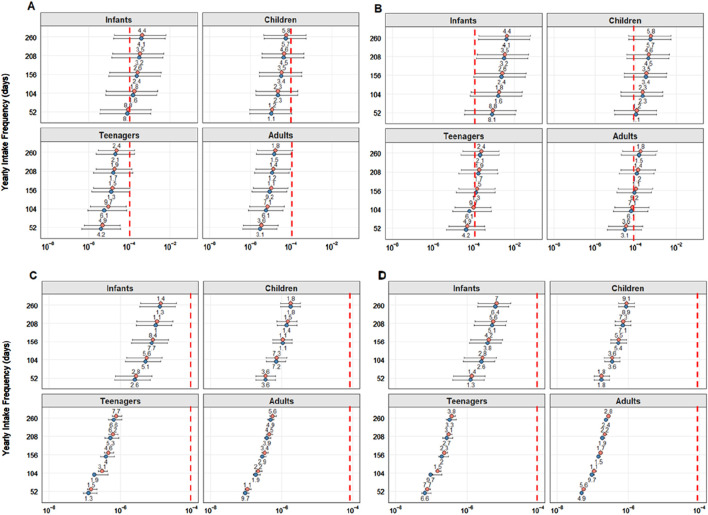
Target cancer risk (TCR) calculated for inorganic arsenic contents of 1% As **(A)** and 10% **(B)**. Cadmium (Cd) **(C)** and lead (Pb) **(D)** per age group and consumption frequencies. The pink and blue dots represent the values for female and male subjects, respectively. The red dashed line represents the established threshold indicating health risks (1 × 10^−4^).

The TCR results for As 1%, As10%, Cd, and Pb results for each assessed age class at 1–5 weekly intake frequencies for both sexes are displayed in Figure 5.

For As 1%, even at the lowest ingestion frequency of once a week, the maximum (indicating the worst-case scenario, calculated using the most contaminated specimen) was above the limit established by the EPA (1 × 10^−4^) (USEPA, 2012) for all age groups and both sexes. When evaluating the 10% scenario, average values ​​were also above the limit for all frequencies, in both sexes, and for babies, children, and adolescents.

Da Silva et al. (2025) reported the following TCR values: 4.84 × 10^−2^ for *Dasyatis* spp., 6 × 10^−3^ for Caribbean sharpnose sharks, and 7.75 × 10^−2^ for *Z. brevirostris* for men and 5.41 × 10^−2^, 6.7 × 10^−3^, and 8.66 × 10^−2^, respectively, for women, confirming higher risks for female subjects, similar to that reported in the present study. Da Silva et al. (2025), however, reported a consumption frequency of 7 days per week (365 days per year), while the present study calculated a more realistic maximum frequency of 5 days per week (260 days per year), resulting in lower TCR values.

In contrast to what was reported by Maciel et al. (2021), in which TCR values for As 10% in Caribbean sharpnose sharks were low, below the established limit (averaging 3.94 × 10^−9^ and calculated as 7.38 × 10^−9^ for the worst-case scenario), while in the present study, even 1% fractions exceeded the limit for some age groups.

For TCR-Cd, the average concentrations were below the carcinogenic risk limit for all sexes, parameters, and age groups. The average value closest to the risk was observed for baby girls ingesting fish five times per week, which was 1.39 × 10^−5^. For the worst-case scenario, calculated based on the most contaminated angular angelshark specimen, with at the highest concentration consumption frequency of 5 days per week, was 3.78 × 10^−5^ for infant girls, still within the acceptable limit.

Regarding the TCR-Pb, no age group surpassed the limit when assessing all consumption frequencies and age groups, even when evaluating the most contaminated specimen. The lowest average TCR-Pb values were found for adult men consuming angular angelshark meat once a week (4.85 × 10^−8^), and the highest values were observed for baby girls consuming this species five times per week (6.96 × 10^−6^). In the worst possible scenario, the most contaminated specimen was noted as comprising a risk for baby girls consuming angular angelshark meat five times a week (1.67 × 10^−5^).

## Conclusion

This study presents the first data on metal contamination in angular angelsharks captured along the coast of Rio de Janeiro, reporting several elements in this species for the first time worldwide. Arsenic concentrations exceeded the safety limits established by the Brazilian ANVISA agency, while other elements like Ti and Rb were detected at levels higher than those reported in comparable species, although still lacking official regulatory thresholds. Although not all consumption parameters indicated immediate concern, the determined Ti and Rb concentrations may pose potential long-term health risks, particularly for vulnerable populations such as infants and women.

The metal and metalloid concentrations detected in the muscle of angular angelsharks were mostly lower than those recorded in other benthic Squatinidae family species, except for Pb and Rb, which stood out as relatively high. The positive balance observed between Se and Hg suggests a potential protective effect of the former against the latter, although this may be canceled out by the presence of other toxic contaminants that indicate significant human health risks, especially for vulnerable groups such as infants and children. Daily and weekly angular angelsharks intake estimates indicated that Cd and MeHg exceeded safe limits for all age groups, representing actual risks, while Cu, Se, and Zn were present in potentially concerning levels in several cases. The calculated non-carcinogenic risk indices (THQ and HI) indicate that As 1%, As 10%, Cu, MeHg, and Se exceeded safe limits, with the 10% inorganic As fraction as the most worrying, especially for female infants, even at reduced consumption frequencies.

Furthermore, even considering a lower consumption frequency, the maximum HI values for iAs 10% exceeded the safety limits for all age groups, reinforcing the seriousness of public health risks associated to angular angelshark meat ingestion in Rio de Janeiro. The risk levels identified in this study were, in fact, substantially higher than that in previous studies, especially regarding As and MeHg, with heightened vulnerability of younger individuals. The CR data indicate that only As consistently exceeded the acceptable limit, with higher values observed in infants, especially female subjects, while the risks associated with Cd and Pb remained within safe limits, representing no immediate risk according to current guidelines. In this sense, we recommend that Brazilian food safety agencies adopt standardized methodologies for human health risk calculations in marine food products, following internationally recognized frameworks. This should, however, also include different assumptions for age groups, body weights, ingestion rates, and inorganic arsenic fractions to enable accurate inter-study comparisons and regulatory decision-making. In the case of angular angelsharks, immediate inclusion of this species in regional contaminant monitoring programs is recommended, with special advisories for vulnerable populations such as infants and pregnant women.

Some limitations are, however, noted as the study is based on samples collected in a single Brazilian state and at a single time-point, which may not capture seasonal or regional variation in metal concentrations. The carcinogenic and non-carcinogenic risk assessments relied on average concentration values per group and literature-derived assumptions (*e.g.,* inorganic As fractions), which may differ from actual exposure scenarios. Future studies should incorporate larger sample sizes, multiple seasons, and direct speciation analyses of arsenic to refine the risk estimates. The interchangeability applied for CR and TCR is also an issue as they are not the same and indicate different risks, thus also requiring standardization.

## Data Availability

The raw data supporting the conclusions of this article will be made available by the authors, without undue reservation.
