# Supplementary material for: Danger on the plate: human health risks derived from the consumption of angular angelshark (Squatina guggenheim) meat in southeastern Brazil
Source: Front Toxicol. 2025 Oct 17;7:1645858. doi: 10.3389/ftox.2025.1645858 (PMC12575100; doi:10.3389/ftox.2025.1645858)
Supplement: Supplementary file 1 [file Table1.docx]

**Supplementary material**

**Supplementary Table 1.** Limits of Detection (LOD) and Limits of Quantification (LOQ) for each investigated element in Groovebelly stingray (Dasyatis hypostigma) specimens sampled from Rio de Janeiro, Southeastern Brazil.

| **Element** | **LOD** | **LOQ** |
| --- | --- | --- |
| **As** | 0.0014 | 0.0047 |
| **Cd** | 0.0009 | 0.0029 |
| **Co** | 0.0003 | 0.0009 |
| **Cu** | 0.004 | 0.015 |
| **Fe** | 0.42 | 13.9 |
| **Hg** | 0.002 | 0.008 |
| **Mn** | 0.0011 | 0.0038 |
| **Pb** | 0.0013 | 0.0045 |
| **Rb** | 0.0006 | 0.0018 |
| **Se** | 0.02 | 0.07 |
| **Ti** | 0.023 | 0.078 |
| **V** | 0.005 | 0.0018 |
| **Zn** | 0.03 | 0.1 |
